# Preparation and *In Vitro* Evaluation of Tacrolimus-Loaded Ethosomes

**DOI:** 10.1100/2012/874053

**Published:** 2012-05-02

**Authors:** Guiling Li, Chao Fan, Xinru Li, Yating Fan, Xiaoning Wang, Mei Li, Yan Liu

**Affiliations:** ^1^Institute of Medicinal Biotechnology, Peking Union Medical College and Chinese Academy of Medical Sciences, Beijing 100050, China; ^2^Department of Pharmaceutics, School of Pharmaceutical Sciences, Peking University, Beijing 100191, China

## Abstract

The main objective of the present work was to prepare and assess dermal delivery of tacrolimus-loaded ethosomes versus classic liposomes. Both delivery systems were characterized for particle size, polydispersity index, and entrapment efficiency (EE), by dynamic laser diffraction and ultrafiltration or dialysis methods, respectively. The results indicated that presence of ethanol in the formulations affected the particle size. In addition, ultrafiltration method was selected to determine EE due to relatively short period as compared with dialysis method. Ethosomes exhibited a significant higher EE and amount of drug in dermis in contrast to classic liposomes suggesting that ethosomes with higher entrapment capacity prompted more amount of tacrolimus to permeate through stratum corneum and reach the target of atopic dermatitis (AD). Physical stability was very well for tacrolimus-loaded ethosomes under storage condition (4°C). Our results demonstrated that the ethosomal system might be a promising candidate for dermal delivery of tacrolimus for AD.

## 1. Introduction

Tacrolimus (C_44_H_69_NO_12_; MW: 822.05) with a 23-member macrolide lactone is discovered from *Streptomyces tsukubaensis*. As a potent immunosuppressor, tacrolimus inhibits the enzyme calcineurin phosphatase which causes an inhibition of transcription of the IL-2 gene, leading to prevention of the T-lymphocyte response [1–3] without some side effects of steroids including cutaneous atrophy, striae, and adrenal suppression [4]. Based on this mechanism, tacrolimus has proven to be efficient for the therapy of either atopic dermatitis (AD) [5, 6] or psoriasis [7–9]. Protopic ointment (Astellas Pharma, USA), the first commercial product of tacrolimus launched for the therapy of AD, was approved in 2002. However, this commercial ointment was reported to be related with high individual variation in mean disposition half-life (*t*_1/2_) [10], the skin irritation such as itching and skin burning with subsequent redness and pain [11], unacceptable greasiness or stickiness and not being water washable with subsequent uneasy feel to the patients, and the finite ability to release drug to the skin because of the intrinsic property of ointment vehicles [12]. More importantly, currently available commercial formulation does not insure adequate topical delivery of the drug into deeper skin layers when applied on skin lesions due to the fact that the main target for tacrolimus is the dermis with its lymphocytes. Therefore, it is highly challenging to overcome the skin barrier stratum corneum (SC) and reach the dermis in sufficient active drug concentrations. These findings prompt for development of novel dermal delivery system permeating the skin barrier and reaching effectively the site of AD.

As an attractive and innovatory vesicular system, ethosome has appeared in the field of drug delivery in recent years [13]. Presenting attractive features correlated with its high deformability, ethosomes could transport active agents more effectively through the stratum corneum into the deeper layers of the skin than classical liposomes [13], which was critically important to the vector applied topically for either topical or systemic drug administration. Beside these, the ethosomal carrier was also capable of providing an efficient intracellular delivery of both hydrophilic and lipophilic molecules [14] and the permeation of an antibiotic peptide [15].

Consequently, the aim of the present study was to develop topical ethosome of tacrolimus and to evaluate it with respect to various *in vitro* experiments. In this paper, ethosomes with phospholipids and ethanol were prepared and evaluated for particle size, polydispersity index (PDI), and drug entrapment efficiency (EE) to investigate the potential application of ethosomes for dermal delivery of tacrolimus. The percutaneous permeation of tacrolimus-loaded ethosomes through SC and epidermis membranes was evaluated *in vitro* and was compared with those of drug-loaded classical liposomes. Further, the stability of tacrolimus-loaded ethosomes was investigated.

## 2. Materials and Methods

### 2.1. Chemicals and Reagents

Lipoid S 100 containing more than 94% phosphatidylcholine from soybean lecithin was purchased from Lipoid Co (Ludwigshafen, Germany). Tacrolimus powder was provided from Taishan Chemical Pharmaceutical Co., LTD (Taishan, China). Protopic ointment was purchased from Astellas Pharma Manufacturing, Inc. (Grand Island, NY, USA). All other chemicals were of analytical grade and used as received.

### 2.2. Animals

 Sprague-Dawley (SD) rats weighing 200 ± 20 g were obtained from Animals Center of Peking University Health Science Center. All care and handling of animals were performed with the approval of Institutional Authority for Laboratory Animal Care of Peking University and followed the principles in the Declaration of Helsinki.

### 2.3. Preparation of Tacrolimus Ethosomes and Liposomes

Ethosomes were prepared from 2% w/v Lipoid S 100, 30% v/v ethanol, 0.1% w/v tacrolimus, and water as described previously [13]. Briefly, Lipoid S 100 was added into a glass vial and solubilized with ethanol. The glass vial was sealed up completely and connected with a tube to a syringe system to allow the addition of water and to avoid ethanol evaporation as far as possible. Following the solubilization of lipoid, water was added to obtain the ethosomal colloidal suspensions, which was agitated for almost 5 min at 50°C. Liposomes loading tacrolimus were prepared by the conventional thin-film hydration method. Generally, Lipoid S 100 for final concentration of 2% w/v and tacrolimus were dissolved in methylene chloride, respectively. Drug was added to furnish the desired concentration in the final formulation (0.1%, w/v). Then organic solvent was removed by rotary evaporation vacuum, and deposited lipid film was hydrated with water by rotation (nearly 100 rpm) for 30 min at room temperature. Finally, liposomal suspensions were sonicated in a bath-type sonicator for 20 min at 5°C for particle homogenization, and then the optically clear suspension was filtered through a 0.22 mm Millipore filter for three cycles.

### 2.4. Particle Size Distribution

For the ethosomal colloidal suspension, the mean size and the polydispersity index (PDI) used as a parameter of the size distribution were measured by dynamic laser light scattering (DLS) with a helium-neon laser at 630 nm (Zetasizer, Malvern, UK). In order to avoid multiscattering phenomena the samples were filtered through 0.45 *μ*m membrane filters. The determination was repeated three times per sample.

### 2.5. HPLC Assay for the Determination of Tacrolimus Content

Quantitative analysis of tacrolimus in ethosomal and liposomal formulations was performed by HPLC. An ODS-Hypersil C18 (250 mm × 4.6 mm i.d., 5-*μ*m particle size) chromatographic column was used. The mobile phase was a mixture of acetonitrile/water (7 : 3, v/v). HPLC apparatus (Shimadzu, Tokyo, Japan) was performed isocratically at 60°C and a flow rate of 1 mL/min with UV detection at 215 nm. Every sample was filtered through a 0.45 *μ*m membrane filter prior to analysis.

### 2.6. Entrapment Efficacy of Ethosomes

The drug-entrapment percentage was determined by two methods: ultrafiltration and dialysis method. At first, unbound tacrolimus was isolated by ultrafiltration on Amicon MPS (Millipore, USA) units using the YM-10 membranes (Millipore, USA) of 10 kDa molecular mass cutoff. 400 *μ*L of ethosomal or liposomal solution was put into a sample compartment of the ultrafiltration unit. Then the unit was centrifuged using a TCL-16G Centrifuger (Shanghai, China) at 10,000 rpm (14,000 ×g) after the attachment of an ultrafiltrate collection container. The filtrate was taken out, and the drug amount was determined by HPLC. The entrapment efficiency (EE) was calculated with the following equation:

(1) $$\frac{D_{E}-D_{S}}{D_{E}}\times 100,$$

where *D*_*E*_ was the amount of tacrolimus determined in the ethosome or liposome and *D*_*S*_ was the amount of drug determined in the filtrate. The results were expressed as a mean value of three times.

At the same time, the drug EE determination was also determined by dialysis method. Cellulose acetate membranes (MWCO 12,000–14,000) were kept into 30% v/v alcoholic solution for 1 h before dialysis to ensure the whole wetting of the membrane; 2 mL of the drug-loaded ethosomes were placed into the dialysis bag which was then transferred into 30 mL of 30% v/v alcoholic solution. Samples of 1 mL were withdrawn from the receiver medium stirred with a magnetic stirrer and replaced with equal volumes of alcoholic solution. Samples were analyzed by HPLC. In this case, the EE was calculated from the same equation described above based on the result analysed by HPLC.

### 2.7. Quantification of Drug in Different Skin Strata

Skin samples were obtained from male SD rats weighing 180 to 220 g. After hair was shaven using a mechanical hair clipper, a 3 × 3 cm patch of skin was cut off from the abdominal region of rat without damaging skin, and then the adhering fat and visceral tissue were removed carefully. The excised rat skin was stored at −20°C and used within one week after harvesting. Skin permeation studies were performed using Franz-type diffusion cells [16] with a diffusional surface area of 2.5 cm^2^ and a volume of 12 mL, respectively. Skin was mounted horizontally with the stratum corneum side up onto the donor compartment. The receptor was filled with pH 7.4 isotonic PBS continuously stirred with a magnetic stirrer in order to ensure its homogeneity. Each formulation (2 mL) was applied in the donor compartment sealed with parafilm in order to obtained occlusive conditions; the amount was sufficient to maintain steady state conditions. The time of duration for the experiments was 48 h. At the end of 48 h the excess formulation from the surface of the skin was absterged. The stratum corneum (SC) layer from the skin was removed by stripping with an adhesive tape, and the epidermis was separated from the dermis using heat separation technique [17]. The tacrolimus in each of the skin layers SC and epidermis was extracted with methanol, and the amount of drug was quantified in the same method as HPLC.

### 2.8. Stability of Ethosomes under Storage Condition

Tacrolimus-loaded ethosomes (0.1%, w/v) were displaced into the amber glass and were preserved at 4°C for 90 days. At predetermined time intervals, samples were transferred into a clear test tube for visual observation; the stability of samples was monitored by the time-dependent changes in the physical characteristics, particle size, and EE during the storage period. The experiments were performed in three replicates.

### 2.9. Statistical Analysis

All data were expressed as mean ± standard deviation (SD) unless particularly outlined. The statistical significance of differences among more than two groups was determined by one-way ANOVA using the software SPSS for Windows versions 13.0. A value of *P* < 0.05 was considered to be significant.

## 3. Results

In the present study, the properties of tacrolimus-loaded ethosomes in terms of size, structure, entrapment efficiency (EE), and amount of tacrolimus in different skin strata in comparison to tacrolimus-loaded liposomes were examined.

### 3.1. Preparation and Characteristics of Ethosomes

**Figure 1:**
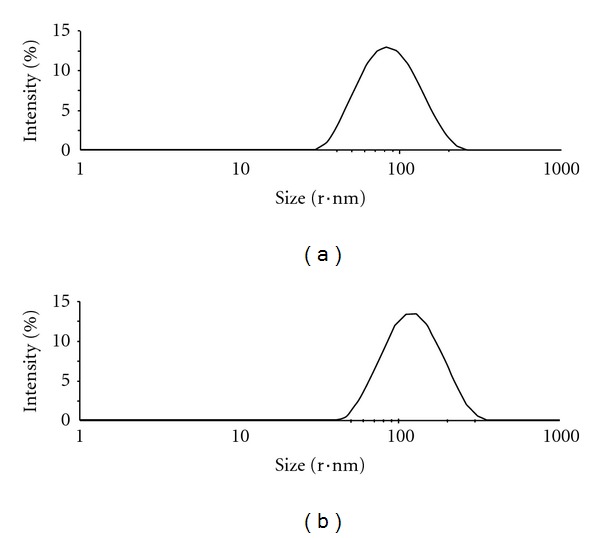
Particle size distribution of tacrolimus liposomes (a) and ethosomes (b) by dynamic laser light scattering.

Particle size affected topical drug delivery; drug-loaded formulation with average diameters below 300 nm could be delivered to some extent into deeper layers of the skin [18, 19]. In the cases of ethosomes and liposomes with particle size lower than 200 nm, there was the potential for permeation through the skin. It was shown from Table 1 and Figure 1 that the particle size of the ethosomal system was significantly smaller than that of the liposomal system (*P* < 0.05). Moreover, polydispersity index (PDI) representing the distribution of particle size was below 0.3 indicating narrow size distribution.

Zeta potential including its ionic atmosphere was the electric potential of the vesicle. Blank ethosomes exhibited a negative charge of −4.3 mV, while the addition of only 0.1% w/w tacrolimus shifted the vesicle charge from negative to positive (+4.8 mV). Further increase of the tacrolimus concentration in the ethosomal system caused a corresponding increase in vesicular charge (data not shown). In comparison, 0.1% w/w tacrolimus had no effect on the charge of classic liposomes.

**Figure 2:**
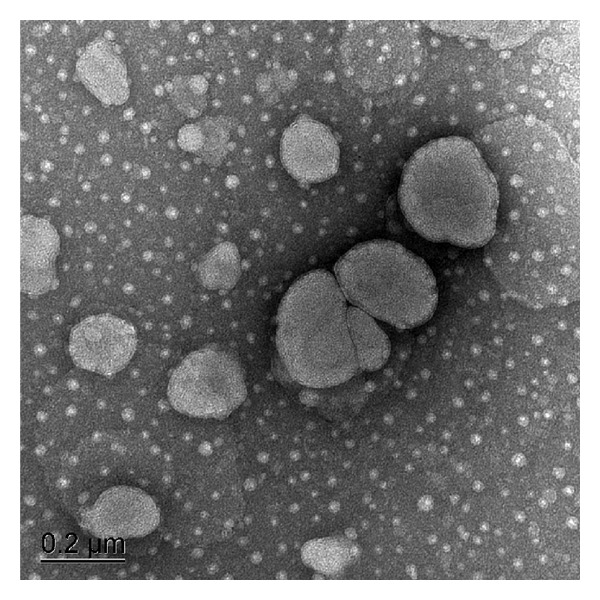
Transmission electron microphotography of tacrolimus ethosomes.

For an initial vesicle characterization, ethosomes was examined by TEM. In all cases, the presence of spherical-shaped vesicles was prominent (Figure 2). Moreover, samples exhibited some vesicle aggregates in the vision field. The cause was that the absence of cholesterol in the formulations was beneficial to the aggregation process among the structures which was proved by other studies [20, 21], on account of cholesterol effect as stabilizing agent in the lipid bilayer.

### 3.2. Determination of the Tacrolimus Content by HPLC

The standard curve of tacrolimus in methanol was shown to be linear (*r* ^2^ = 0.9993) over the concentration range 0.02–1.50 *μ*g/mL. The following regression equation was obtained: *A* = 12697*C* − 6615, where *A* is the peak area of tacrolimus and *C* is the concentration of tacrolimus (*μ*g/mL). Prior to the study, all related blank samples (alcoholic solution, lipoid S100 in methanol) were analyzed by HPLC. The result indicated that the retention time of tacrolimus chromatograph was 15 min under this condition, while the resulting HPLC chromatograms of alcoholic solution and lipoid S 100 showed no peaks eluted from 14 to 15 min. Thus, there was no interference on the determination of tacrolimus content by HPLC caused by these blank samples. Besides, accuracy ranged from 101.2% to 105.0% for all samples, and precision was below 2.0, indicating that the system was reliable for analysis. The recovery range and RSD for all samples were found to be 98.0%–101.5% and 1.1%–1.7%, respectively. Thus, acceptable results with respect to precision (RSD < 2%) were obtained for all analytes.

### 3.3. Entrapment Efficiency

There were two different methods such as ultrafiltration and dialysis used to quantify the tacrolimus amount entrapped into the systems. EE determined with the ultrafiltration method was (78.7 ± 3.3)% which was slightly lower than that with the dialysis method (80.4 ± 4.4)%. The result suggested a losing of entrapped drug during the ultrafiltration process probably due to the deformation phenomenon of lipid membranes caused by the high speed reached with this method. As a result, based on no significant difference in EE between above two methods (*P* > 0.05), ultrafiltration method was chosen due to longer period for dialysis method.

As shown in Table 1, ethosomes and liposomes exhibited an entrapment efficiency of about 78.7% and 65.3%, respectively, indicating that solubility of the drug in the medium might influence the entrapment capacity of vesicles. In liposomes, tacrolimus as a highly lipophilic molecule was only in the bilayer and did not enter the aqueous core. It was assumed that the core and membrane of the ethosome were saturated with medium, which might allow for tacrolimus to be distributed throughout the vesicle, as opposed to only the bilayer with liposomes.

### 3.4. Quantification of Drug in Different Skin Strata

**Figure 3:**
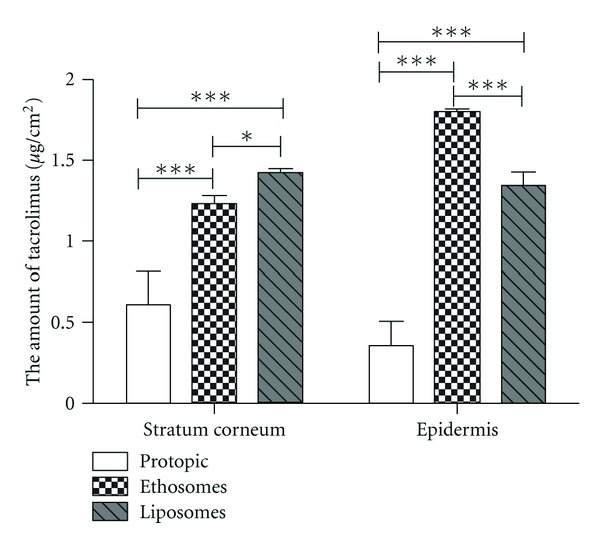
The amount of tacrolimus in stratum corneum and epidermis for tacrolimus formulations.

As shown in Figure 3, the amounts of tacrolimus for ethosomes and liposomes were significantly higher than that for Protopic ointment whether in SC or epidermis (*P* < 0.001), suggesting that two vesicular formulations might improve drug distribution in deeper skin with AD. Relatively, the amount of tacrolimus for ethosomes was lower than that for liposomes in SC (*P* < 0.05) and was significantly higher than that for liposomes in epidermis (*P* < 0.001), indicating that more amount of drug within ethosomes would permeate into more deeper epidermis as compared with liposomes, which suggested stronger pharmacological effect for ethosomes.

### 3.5. Stability under Storage Condition

The stability of investigated systems was determined by comparing the physical appearance, average size, PDI, and EE over time.  Table 2 presented the stability of ethosomes kept at 4°C and measured at various time intervals after preparation. It was shown that tacrolimus-loaded ethosomal system resulted in a slight increase of particle size and PDI at 3 months (*P* > 0.05, versus 0 month), and neither precipitation nor drug crystals were observed, indicating that the ethosomes were still intact. Moreover, EE did not alter significantly after storage for three months attributed to that the oxidation and hydrolysis of the lipids caused in decomposition and aggregation of the liposome vesicles, drug leakage, and slight decrease in the zeta potential at room temperature were inhibited at low temperature.

## 4. Discussion

According to the physicochemical characteristics of tacrolimus-loaded vesicular formulations (particle size and PDI), as shown in Table 1 and Figure 1, it was suggested that ethanol had a fluidizing effect on phospholipid bilayers [13, 22] as compared with common liposomes. Furthermore, ethanol probably caused an alteration of the net charge of the system and conferred it some extent of steric stabilization that might finally lead to a reduction in the mean particle size [23, 24]. In other words, ethanol had a predominant role in ethosomal systems since introducing ethanol to conventional liposomes produces tender, compact, and transformable vesicles [22].

Ethosomal systems with interdigitated fluid bilayers are novel permeation-enhancing lipid carriers. It has been shown that ethosomes exhibit high encapsulation efficiency for a series of lipophilic molecules and deliver effectively drugs to and through the skin [13, 25, 26] in contrast to classic liposomes. The results (Figure 3.) showed that the drug encapsulation within ethosomes or liposomes could influence positively the permeation of a molecule through SC to some degree, although the real contribution was difficult to evaluate due to the deformable and permeable nature of ethosomes which allowed the rapid drug diffusion from the environment into ethosome compartments. These findings might be attributed to some reasons as below: first, the stratum corneum (SC) lipid multilayers were highly conformationally ordered and densely packed at physiological temperature. Ethanol might interact with lipid molecules in the polar headgroup region inducing a reduction in the *T*_*m*_ of the SC lipids. Thus the organization of the SC lipid bilayer was disturbed, and their fluidity was enhanced. Second, the intercalation of ethanol into the polar headgroup environment could lead to an increase in the membrane permeability [27, 28]. That is to say, ethosomal lipids were in a more fluid state than liposomes containing the same ingredients without ethanol. These data are supported by previous reports that ethanol generally has a fluidizing effect on phospholipid bilayers [29]. In contrast to liposomes, ethosomes are less rigid [30]. Therefore, the effects of ethanol might give the vesicles soft flexible characteristics to more easily penetrate into deeper layers of the skin, although it was considered harmful to classic liposomal formulation. The third contribution to the high skin penetration from the ethosomal system could have connection with the interaction between ethanol together with phospholipid vesicles and SC which was also proposed by Kirjavainen et al. [31]. It has also been suggested that mixing of phospholipids with SC lipids of the intercellular layers enhanced the permeability of the skin [32]. In summary, the effect of ethanol on SC lipids and on vesicle fluidity as well as a dynamic interaction between ethosomes and SC might contribute to the superior delivery properties described here. While tacrolimus encapsulated in classic ointment remained primarily at the surface of the skin, the ethosomal system was shown here to be a highly effective carrier for enhanced tacrolimus delivery to deeper layers of the skin.

## 5. Conclusion

In this paper, liposome and ethosome formulations containing tacrolimus as a lipophilic drug have been prepared successfully and characterized. It was observed that the presence of ethanol in the aqueous compartment of the vesicles favoured tacrolimus encapsulation. Therefore, the inclusion of ethanol in liposomes might play a vital role in the enhancement of tacrolimus permeation. The efficient drug delivery shown here together with the long-term stability of ethosomes made this system a promising candidate for topical delivery of tacrolimus. Currently, further studies involving safety on skins, pharmacological effect on AD, and mechanism of inhibition action for the ethosomal delivery systems were being performed.

## Figures and Tables

**Table 1:** Physicochemical characteristics of tacrolimus-loaded liposomes and ethosomes.

	Liposomes	Ethosomes
Particle size (nm)	146 ± 12	85 ± 13*
Polydispersity index	0.268 ± 0.051	0.298 ± 0.024
Entrapment efficiency (%)	65.3 ± 2.2	78.7 ± 3.3**

*P < 0.05; **P < 0.01 versus liposomes.

**Table 2:** The stability of tacrolimus-loaded ethosomes under storage condition (4°C).

	0 month	3 month
Particle size (nm)	85 ± 13	96 ± 11^ns^
Polydispersity index	0.298 ± 0.024	0.312 ± 0.031^ns^
Entrapment efficiency (%)	78.7 ± 3.3	74.3 ± 4.5^ns^

^ns^ *P* > 0.05 versus 0 month.

## References

[1] Thomson AW, Bonham CA, Zeevi A (1995). Mode of action of tacrolimus (FK506): molecular and cellular mechanisms. *Therapeutic Drug Monitoring*.

[2] Kelly PA, Burckart GJ, Venkataramanan R (1995). Tacrolimus: a new immunosuppressive agent. *American Journal of Health-System Pharmacy*.

[3] Wohlrab J (2006). Calcineurin inhibitors for topical therapy in psoriasis. *Der Hautarzt*.

[4] Rubins A, Gutmane R, Valdmane N, Stevenson P, Foster C, Undre N (2005). Pharmacokinetics of 0.1% tacrolimus ointment after first and repeated application to adults with moderate to severe atopic dermatitis. *Journal of Investigative Dermatology*.

[5] Carroll CL, Fleischer AB (2006). Tacrolimus: focusing on atopic dermatitis. *Drugs of Today*.

[6] Fleischer AB, Abramovits W, Breneman D, Jaracz E (2007). Tacrolimus ointment is more effective than pimecrolimus cream in adult patients with moderate to very severe atopic dermatitis. *Journal of Dermatological Treatment*.

[7] Kroft EBM, Erceg A, Maimets K, Vissers W, van der Valk PGM, van de Kerkhof PCM (2005). Tacrolimus ointment for the treatment of severe facial plaque psoriasis. *Journal of the European Academy of Dermatology and Venereology*.

[8] Lebwohl M, Freeman A, Chapman MS, Feldman S, Hartle J, Henning A (2005). Proven efficacy of tacrolimus for facial and intertriginous psoriasis. *Archives of Dermatology*.

[9] Steele JA, Choi C, Kwong PC (2005). Topical tacrolimus in the treatment of inverse psoriasis in children. *Journal of the American Academy of Dermatology*.

[10] Cheer SM, Plosker GL (2001). Tacrolimus ointment: a review of its therapeutic potential as a topical therapy in atopic dermatitis. *American Journal of Clinical Dermatology*.

[11] Rustin MHA (2007). The safety of tacrolimus ointment for the treatment of atopic dermatitis: a review. *British Journal of Dermatology*.

[12]  Kudla RM Topical ointment.

[13] Touitou E, Dayan N, Bergelson L, Godin B, Eliaz M (2000). Ethosomes—novel vesicular carriers for enhanced delivery: characterization and skin penetration properties. *Journal of Controlled Release*.

[14] Touitou E, Godin B, Dayan N, Weiss C, Piliponsky A, Levi-Schaffer F (2001). Intracellular delivery mediated by an ethosomal carrier. *Biomaterials*.

[15] Godin B, Touitou E (2004). Mechanism of bacitracin permeation enhancement through the skin and cellular membranes from an ethosomal carrier. *Journal of Controlled Release*.

[16] Li X, Li X, Zhou Y (2010). Development of patch and spray formulations for enhancing topical delivery of sinomenine hydrochloride. *Journal of Pharmaceutical Sciences*.

[17] Kikwai L, Babu RJ, Prado R (2005). In vitro and in vivo evaluation of topical formulations of Spantide II. *AAPS PharmSciTech*.

[18] du Plessis J, Ramachandran C, Weiner N, Müller DG (1994). The influence of particle size of liposomes on the deposition of drug into skin. *International Journal of Pharmaceutics*.

[19] Verma DD, Verma S, Blume G, Fahr A (2003). Particle size of liposomes influences dermal delivery of substances into skin. *International Journal of Pharmaceutics*.

[20] Coderch L, Fonollosa J, De Pera M, Estelrich J, De La Maza A, Parra JL (2000). Influence of cholesterol on liposome fluidity by EPR: relationship with percutaneous absorption. *Journal of Controlled Release*.

[21] Brisaert M, Gabriëls M, Matthijs V, Plaizier-Vercammen J (2001). Liposomes with tretinoin: a physical and chemical evaluation. *Journal of Pharmaceutical and Biomedical Analysis*.

[22] Touitou E, Godin B, Weiss C (2000). Enhanced delivery of drugs into and across the skin by ethosomal carriers. *Drug Development Research*.

[23] Lasic D, Weiner N, Riaz M, Martin F (1998). Liposomes. *Pharmaceutical Dosage Forms: Disperse Systems*.

[24] López-Pinto JM, González-Rodríguez ML, Rabasco AM (2005). Effect of cholesterol and ethanol on dermal delivery from DPPC liposomes. *International Journal of Pharmaceutics*.

[25] Touitou E Composition for applying active substances to or through the skin.

[26] Touitou E Compositions for applying active substances to or through the skin.

[27] Berner B, Liu P (1995). Alcohols. *Percutaneous Penetration Enhancers*.

[28] Komatsu H, Okada S (1996). Ethanol-enhanced permeation of phosphatidylcholine/phosphatidylethanolamine mixed liposomal membranes due to ethanol-induced lateral phase separation. *Biochimica et Biophysica Acta—Biomembranes*.

[29] Harris RA, Burnett R, McQuilkin S, Mcclard A, Simon FR (1987). Effects of ethanol on membrane order: fluorescence studies. *Annals of the New York Academy of Sciences*.

[30] Cevc G, Schätzlein A, Richardsen H (2002). Ultradeformable lipid vesicles can penetrate the skin and other semi-permeable barriers unfragmented. Evidence from double label CLSM experiments and direct size measurements. *Biochimica et Biophysica Acta—Biomembranes*.

[31] Kirjavainen M, Urtti A, Valjakka-Koskela R, Kiesvaara J, Mönkkönen J (1999). Liposome-skin interactions and their effects on the skin permeation of drugs. *European Journal of Pharmaceutical Sciences*.

[32] Blume A, Jansen M, Ghyczy M, Gareiss J (1993). Interaction of phospholipid liposomes with lipid model mixtures for stratum corneum lipids. *International Journal of Pharmaceutics*.

